# How Factors Involved in the Resolution of Crystal-Induced Inflammation Target IL-1β

**DOI:** 10.3389/fphar.2017.00164

**Published:** 2017-03-28

**Authors:** Francesca Oliviero, Anna Scanu

**Affiliations:** Rheumatology Unit, Department of Medicine – DIMED, University of PadovaPadova, Italy

**Keywords:** crystal-induced inflammation, interleukin-1, transforming growth factor, lipoproteins, neutrophil extracellular traps, dietary factors

## Abstract

One of the main clinical features characterizing crystal-induced inflammation is its spontaneous resolution. The aim of this review is to outline the various factors involved in the self-limiting course of crystal-induced inflammation focusing on their effect on IL-1β production. Endogenous molecules that are induced or locally recruited by the process itself, inhibitory proteins naturally present in the joint and exogenous dietary factors are discussed. Aside from the classical well-known molecules involved in the resolution of crystal-induced acute attack such as TGFβ, IL-10, IL-1Ra, and lipoproteins, particular attention is paid to recently uncovered mechanisms such as the aggregation of neutrophil extracellular traps, the release of ectosomes from neutrophil surface, and alpha-1-anti-trypsin-mediated IL-1 inhibition.

## Introduction

Crystal-induced inflammation is caused by the presence of monosodium urate (MSU) or calcium pyrophosphate (CPP) crystals in articular or periarticular tissues. Although MSU and CPP crystals form in different ways, their effects are very similar and associated with an acute, intense inflammatory reaction characterized by massive leukocyte recruitment and the local release of cytokines, chemokines, reactive oxygen species and proteolytic enzymes (Liu-Bryan and Lioté, 2005).

Since the time cytoplasmic NACHT-LRRPYD-containing protein-3 (NLRP) 3 inflammasome was first identified and its activation by MSU and CPP crystals was demonstrated (Martinon et al., 2006), interleukin (IL)-1β has been considered the most important inflammatory mediator in crystal-induced inflammation, and it represents one of the main targets for new drugs that have been or are being developed to treat gout and calcium crystal-induced arthritis (Dinarello, 2014).

Although the molecular mechanisms leading to the activation of NLRP3 by pathogenic crystals have not been fully elucidated, the two-step process linked to the production of IL-1β has been clearly demonstrated. The first signal is triggered by pattern-recognition receptors (e.g., TLRs) which initiate the transcription of IL-1β; the second signal triggers inflammasome activation, which in turn activates caspase-1 and leads to the cleavage of the IL-1β precursors into the active cytokine (Joosten et al., 2010; Netea et al., 2015; Scanu et al., 2016). Once released, the cytokine promotes the induction of different pro-inflammatory genes amplifying the inflammatory process and leading to long term articular damage. Similarly to IL-1β, IL-18 is produced by caspase-1 activation but its role in crystal-induced inflammation has not been clearly defined.

**FIGURE 1:**
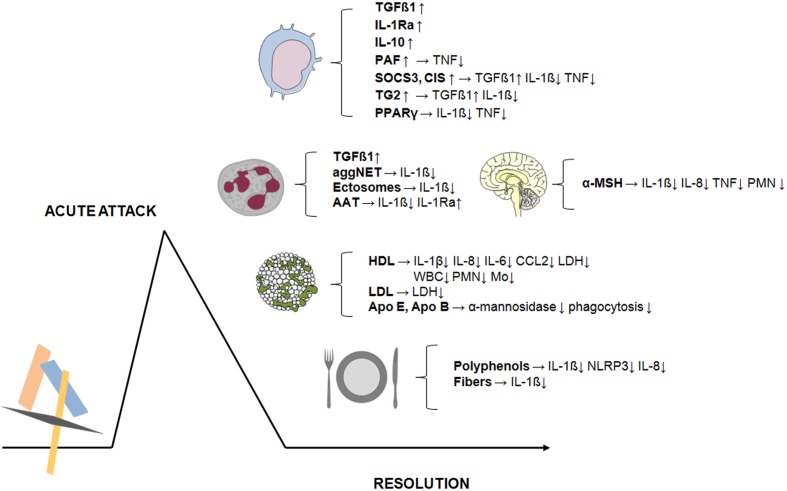
**Endogenous and exogenous factors involved in the resolution of crystal-induced inflammation.** The acute attack triggered in the joints by MSU or CPP crystals resolves in a few days. Various factors are involved in this self-limiting process. Most of these are endogenous molecules (TGFβ1, IL-1Ra, IL-10, PAF, PPARγ) or intracellular negative cytokine regulators (SOCS3, CIS, TG2) induced in monocytes/macrophages following crystal exposure. Others are hormones (α-MSH) or proteins produced by neutrophils (TGFβ1, AAT) or molecules naturally present in the joints (lipoproteins and apoproteins). Dynamic processes involving neutrophils such as aggNET and ectosomes release are also involved in the resolution of crystal-induced inflammation. Finally, exogenous dietary components (phenolic compounds and fibers) play an additional role in modulating the inflammatory process induced by pathogenic crystals. → Lead to the production of; ↑ increase; ↓ decrease.

One of the main clinical features characterizing crystal-induced inflammation is its spontaneous resolution. It is well-known that patients experiencing an acute attack improve within a few days time and that the disease progresses to a chronic state only if untreated (Punzi et al., 2012). The various factors involved in the self-limiting course of crystal-induced inflammation are outlined in this review. Most of these are endogenous molecules that are induced or locally recruited by the process itself or are inhibitory proteins naturally present in the joint. Other factors are exogenous dietary substances that can modulate the resolution of the acute attack (**Figure 1**).

An in-depth analysis of these mechanisms reveals that the final outcome is a direct or indirect action on IL-1β production. In particular, a negative regulation of inflammasome activation and pro-IL-1β expression have been described.

### Transforming Growth Factor

Transforming growth factor (TGF) β1 is one of the main molecules involved in the resolution of crystal-induced inflammation. The effect of TGFβ1 was initially described in a subcutaneous air pouch animal model in which it inhibited MSU crystal-induced leukocyte chemotaxis (Lioté et al., 1996).

Some investigators hypothesized that the shift from a pro-inflammatory state to an active production of anti-inflammatory molecules is the mechanism leading to the resolution of an acute attack (Chen et al., 2011). It has been shown, in fact, that the differentiation of monocytes into macrophages and the uptake of crystals by the latter induces TGFβ1 secretion (Chen et al., 2011). The presence of high levels of TGFβ1, IL-1 receptor antagonist (Ra), IL-10 and soluble receptors of tumor necrosis factor (TNF) has, in fact, been demonstrated in the synovial fluid (SF) of patients with gout and has been associated with the upregulation of intracellular negative cytokine regulators such as the suppressors of cytokine signalling (SOCS)3 and the cytokine inducible SH2-containing protein (CIS). While enhancing TGFβ1 expression, these proteins downregulate IL-1β and TNF secretion (Chen et al., 2011).

In addition, the inverse relationship between TGFβ1 and IL-1β was clearly demonstrated by a study examining different inflammatory mediators in the SF collected during different stages of acute gout attack (Scanu et al., 2012).

A tight regulation of IL-1β production by TGFβ1 has also been demonstrated with regard to neutrophils. Activated neutrophils have, in fact, been identified as an additional potential source of local TGFβ1 production. An increased TGFβ1 expression following neutrophil phagocytosis of apoptotic cells, a process which in turn regulates IL-1β production by the neutrophils themselves, has been described (Steiger and Harper, 2013).

The negative regulation of IL-1β production by the crystal-induced TGFβ1 signaling pathway has been confirmed by other investigators. In particular, a higher transglutaminase (TG) 2 expression in crystal-exposed macrophages has been linked to TGFβ production (Yen et al., 2015) which, in turn, downregulates IL-1β release through the inhibition of the Janus kinase (JAK) 2 signaling. TG is indeed highly expressed in gout and has been shown to enhance phagocytosis of apoptotic leukocytes by macrophages limiting neutrophil accumulation and promoting resolution (Rose et al., 2006).

Importantly, TGFβ1 has been shown to inhibit cell surface IL-1 receptor expression (Dubois et al., 1990) supporting an additional role in limiting crystal-induced inflammatory response.

### Lipoproteins

Several studies have demonstrated the importance of lipoproteins as modulators of crystal-induced inflammation. It has been hypothesized that changes in SF lipoprotein concentration and in the proteins that coat crystals play an integral role in the self-limiting nature of an acute attack. In this context, lipoproteins containing apolipoproteins (apo) B and E, have demonstrated potential regulatory effects suppressing MSU crystal phagocytosis and alpha-mannosidase release by neutrophils (Terkeltaub et al., 1986, 1991). This is probably due to the ability of these proteins to displace complement-activating IgG that initially cover the crystals (Ortiz-Bravo et al., 1993). It has also been hypothesized that the binding of low density (LDL) and high density lipoproteins (HDL) to the crystal surface reduces MSU- and CPP-induced lactate dehydrogenase release from neutrophils, a marker for cytolysis (Burt et al., 1989). It has been shown that the levels of LDL in the rat air-pouch model surge and remain elevated 24 h after the injection of CPP crystals, while the number of white blood cells and the concentration of β-Glucoronidase and PGE2 significantly fell (Kumagai et al., 2001). In addition, it has been reported that HDL inhibit MSU crystal-induced CCL2 production and expression in human synoviocytes and reduce monocyte/macrophage recruitment (Scanu et al., 2010).

Although several studies have demonstrated that lipoproteins may contribute to limiting crystal induced inflammation, their effects on the production of IL-1β have been evaluated only for HDL. Air pouch experiments have shown that HDL display anti-inflammatory activity reducing the recruitment of leukocytes and affecting pro- and anti-inflammatory cytokine balance after MSU crystal injection. It has been reported, in particular, that HDL reduce the release of IL-1β in pouch exudates and the mRNA levels in membranes. Interestingly, HDL do not affect the production of the crystal-induced anti-inflammatory factor IL-1Ra. Unlike other lipoproteins, HDL may act not only by adhering to the surface of the crystals, but also through a direct interaction with the inflammatory cells (Scanu et al., 2015).

### IL-1Ra

Although IL-1Ra is the natural IL-1 inhibitor as it functions as an IL-1 receptor competitor (Dayer, 2002) and its effectiveness in the treatment of gout has been established, few studies have been conducted to assess its role in the spontaneous resolution of crystal-induced inflammation. Anti-inflammatory cytokine assessment in SF demonstrated that IL-1Ra levels are higher in gouty patients than in osteoarthritis patients (Chen et al., 2011). The injection of MSU crystals into a murine air pouch causes IL-1Ra production after 3 h, although the levels detected are not sufficient to inhibit the inflammatory effect of IL-1β at that same time point (Scanu et al., 2015). *In vitro* stimulation of monocytes/macrophages by synthetic MSU, CPP and basic calcium phosphate (BCP) crystals induces a rapid increase in pro-inflammatory cytokines such as IL-1β, IL-8 and IL-6, whereas longer periods are required to release high levels of IL-1Ra (a personal observation).

### Neutrophil Extracellular Traps and Microvesicles

The aggregation of neutrophil extracellular traps (NETs) induced by pathogenic crystals has been recently associated with the resolution of neutrophilic inflammation that characterizes the acute crystal-induced inflammatory process.

NET formation (NETosis) is accompanied by the release of a variety of pro-inflammatory mediators that orchestrate the local innate immune response. When neutrophils are under conditions of high cell density, crystal-induced NETs form dense aggregates that sequester and degrade neutrophil inflammatory mediators, in particular IL-1β. It has been demonstrated that this process is mediated by ROS which, in this particular case, downregulates inflammation (Schauer et al., 2014).

Another phenomenon that is associated with the resolution of crystal-induced arthritis is the release of phosphatidylserine positive ectosomes from the surface of neutrophils during the inflammatory process. It has been demonstrated that these ectosomes suppress inflammasome and consequently inhibit IL-1β release in C5a primed macrophages (Cumpelik et al., 2016). Although ectosomes trigger the release of TGF by monocyte and macrophages, it was not found that TGF was necessary to suppress inflammasome activation.

### Other Endogenous Factors

A variety of other regulatory factors involved in the spontaneous resolution of an acute attack of crystal-induced arthritis have been identified. Among them, peroxisome proliferator-activated receptor γ (PPARγ), a nuclear hormone receptor, has been shown to be expressed in human monocytes after MSU crystal stimulation. PPARγ ligands reduce the crystal-induced production of IL-1β and TNF *in vitro* (Akahoshi et al., 2003).

Similarly, melacortin receptor (MC-R) agonists can also influence the resolution of an acute gout attack. Selective ligands for MC-R3, such as α-melacortin-stimulating hormone (α-MSH), lower the levels of IL-1β and the chemokine (C-X-C motif) ligand 1 (CXCL1) and the polymorphonuclear cell (PMN) migration in a murine model of MSU crystal-induced peritonitis (Getting et al., 2001, 2006). In addition, it was found that α-MSH inhibits, *in vitro*, the capacity of monocytes to release IL-1β and other pro-inflammatory cytokines in response to MSU crystals, without affecting the secretion of caspase-1, the enzyme responsible for converting pro-IL-1β to cytokine’s active form (Capsoni et al., 2009). By contrast, β-hydroxybutyrate (BHB), a ketone body produced in the liver, has been observed to inhibit IL-1β processing in response to MSU crystals by reducing caspase-1 activation. BHB has been shown to block NLRP3 inflammasome preventing the decline of K^+^ intracellular efflux induced by NLRP3 activators (Youm et al., 2015).

Other molecules that may trigger resolution are induced during inflammatory processes promoted by crystals, even though they may play a minor role. Higher levels of IL-10 were observed in the SF from patients with acute gout with respect to OA patients (Chen et al., 2011). But although it has been demonstrated that IL-10 overexpression blocks MSU crystal-induced inflammation, including the suppression of TNF release *in vitro* and CXCL1 production *in vivo* (Murakami et al., 2002), there is no evidence of IL-10 in supernatants from differentiated macrophages incubated with MSU crystals (Yagnik et al., 2004).

Macrophage derived platelet activating factor (PAF) and related molecules could, according to one hypothesis, play a role in suppressing the inflammatory response. *In vitro* experiments demonstrated that MSU crystal-stimulated macrophages release PAF, which in turn downregulates the TNF secretion (Yagnik, 2014).

The anti-inflammatory effect of IL-10 and PAF has not been assessed in the crystal-induced IL-1β production or action/pathway.

A recently described mechanism involved in the inhibition of crystal-induced inflammation concerns alpha-1-anti-trypsin (AAT), the major, natural inhibitor of serine proteases produced by neutrophils that can be found in human serum during infections and inflammation. Serine proteases have been found to be responsible for the caspase-1-independent conversion of IL-1β precursor into the active cytokine in neutrophils (Joosten et al., 2009) which are the main inflammatory cells in crystal arthritis. It has been demonstrated that AAT not only reduces the release and the extracellular processing of IL-1β but also increases circulating levels of endogenous IL-1Ra, the natural inhibitor of IL-1 (Joosten et al., 2016).

### Exogenous Factors

Although, it is well-established that nutrients or dietary metabolites possess immune-regulatory properties and can modulate the inflammatory response (Camell et al., 2015), their role in crystal-induced inflammation has only recently been taken into consideration.

Well-known for their antioxidant, anti-inflammatory and anti-cancer effects, plant polyphenols are the most studied dietary compounds. In particular, green tea epigallocatechin-3-gallate (EGCG) has been observed to reduce the inflammatory response to CPP crystals by inhibiting IL-1β, IL-8, and CCL2 *in vitro* (Oliviero et al., 2013). An inhibitory effect exerted by EGCG was also demonstrated in urate crystal-induced peritoneal inflammation. A reduction in IL-1β levels in the peritoneal lavage fluid has been observed together with a lower NLRP3 inflammasome expression (Jhang et al., 2016).

An inhibition of NLRP3 has also been reported by investigators assessing the anti-inflammatory properties of two other dietary polyphenols, morin (Dhanasekar and Rasool, 2016) and ferulic acid (Doss et al., 2016) in a rat model of acute gout.

Other studies have investigated the beneficial effects of natural compounds in experimental gout models (Martin et al., 2009; Sabina et al., 2011; Huang et al., 2012). Although those studies did not take into full consideration their effect on IL-1β pathway, the overall result was an inhibition of the crystal-induced inflammatory process.

Some interesting findings were produced using butyrate, a short-chain fatty acid produced in the colon by the fermentation of insoluble dietary fibers (Cleophas et al., 2016). This substance has been shown to suppress urate crystal-induced IL-1β production and expression by the inhibition of the histone deacetylase (HDAC) 8, an enzyme that regulates the expression and activity of various proteins. Although the exact mechanism of action is not clear, butyrate did not increase IL-Ra or TGFβ1, classically involved in the resolution phase of the inflammatory response to pathogenic crystals.

Recently, it has been demonstrated that a high-fiber diet and acetate, a short-chain fatty acid resulting from the metabolism of fiber by gut microbiota, induce faster resolution of the inflammatory response triggered in mice after injection of MSU crystals into the knee joint. In particular, acetate promotes caspase-dependent apoptosis of neutrophils associated with reduced NF-kB activity, lower IL-1β tissue concentations, increased production of anti-inflammatory mediators, such as TGF-β, IL-10 and annexin A1, and enhanced efferocytosis (Vieira et al., 2017).

## Conclusion

Several mechanisms seem to be involved in resolving crystal-induced inflammation. Although some are linked to exogenous dietary factors, an acute attack is limited by molecules that are activated or expressed following the attack itself through a finely tuned self-regulating mechanism.

**FIGURE 2:**
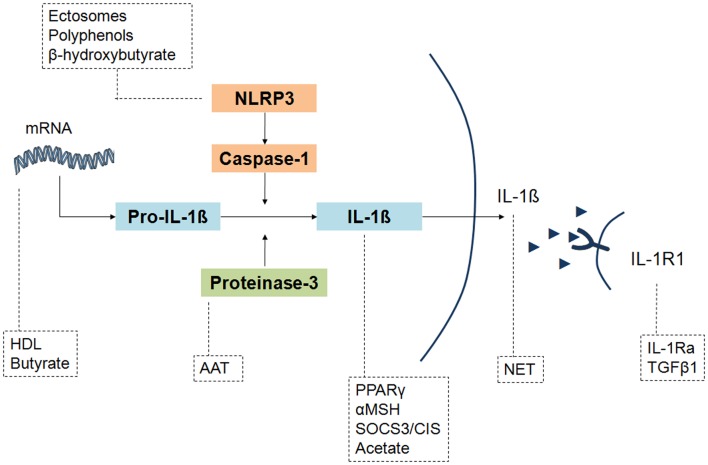
**Different levels of IL-1 inhibition by the endogenous and exogenous factors involved in the resolution of crystal-induced inflammation.** IL-1β production pathway can be affected by molecules that act upstream on NLRP3 activation (ectosomes, polyphenols, β-idroxybutyrate) and IL-1β mRNA expression (HDL, butyrate), or downstream on pro-IL-1β cleavage (AAT), IL-1β formation (PPARγ, αMSH, SOCS3/CIS, acetate) and concentrations (NET), or inhibiting IL-1β bond to its receptor IL-1R1 (IL-1Ra, TGFβ1). Although the final effect of PPARγ, αMSH and acetate is an inhibition of IL-1β levels, their precise mechanism of action has not been elucidated.

As outlined above, these molecules can act at different molecular levels, by affecting cell-crystal interactions, gene expression and cytokine trapping. As regard to IL-1β production process, **Figure 2** evidences the substances involved upstream at the mRNA and inflammasome inhibition level, and downstream at IL-1β production, IL-1β release and IL-1R1 signaling level.

Although the precise mechanism of action of some of these factors has not been fully characterized, the final outcome is a diminished IL-1β production.

Considering the importance of IL-1 blocking agents in reducing acute attacks, firstly noted with regard to IL-1Ra anakinra, and then to the anti-IL-1β monoclonal antibody canakinumab, every molecule capable to reduce IL-1β production could represent a potential therapeutic target and have a positive impact on the clinical practice.

## Author Contributions

All authors listed, have made substantial, direct and intellectual contribution to the work, and approved it for publication.

## Conflict of Interest Statement

The authors declare that the research was conducted in the absence of any commercial or financial relationships that could be construed as a potential conflict of interest.
